# A novel approach to group decision-making using generalized bipolar neutrosophic sets

**DOI:** 10.1371/journal.pone.0317746

**Published:** 2025-06-11

**Authors:** Aliya Fahmi, Aziz Khan, Thabet Abdeljawad

**Affiliations:** 1 Department of Mathematics and Statistics, The University of Faisalabad, Pakistan; 2 Department of Mathematics and Sciences, Prince Sultan University, Riyadh, Saudi Arabia; 3 Department of Mathematics, Saveetha School of Engineering, Saveetha Institute of Medical and Technical Sciences, Saveetha University, Chennai, Tamil Nadu, India; 4 Department of Medical Research, China Medical University, Taichung, Taiwan; 5 Center for Applied Mathematics and Bioinformatics (CAMB), Gulf University for Science and Technology, Hawally, Kuwait; 6 Department of Mathematics and Applied Mathematics, Sefako Makgatho Health Sciences University, Garankuwa, Medusa, South Africa; 7 Department of Mathematics, Kyung Hee University 26 Kyungheedae-ro, Dongdaemun-gu, Seoul, Korea; Sefako Makgatho Health Sciences University Faculty of Health Sciences, SOUTH AFRICA

## Abstract

This study introduces operational laws for Aczél-Alsina aggregation within the framework of generalized bipolar neutrosophic sets (GBNS), tailored for group decision-making scenarios. Novel aggregation operators, including the Generalized Bipolar Neutrosophic Aczél-Alsina Weighted Average (GBNAAWA), Generalized Bipolar Neutrosophic Aczél-Alsina Ordered Weighted Average (GBNAAOWA), Generalized Bipolar Neutrosophic Aczél-Alsina Hybrid Weighted Average (GBNAAHWA), Generalized Bipolar Neutrosophic Aczél-Alsina Weighted Geometric (GBNAAWG), Generalized Bipolar Neutrosophic Aczél-Alsina Ordered Weighted Geometric (GBNAAOWG), and Generalized Bipolar Neutrosophic Aczél-Alsina Hybrid Weighted Geometric (GBNAAHWG), are proposed to address complex decision-making processes under uncertainty. The methodology is demonstrated through a case study and an illustrative example to validate its practical applicability. Comparative and sensitivity analyses highlight the robustness and adaptability of the proposed operators in various decision contexts. Key findings, discussions, and limitations are presented to provide insights into the method’s effectiveness and areas for future research. This work contributes to advancing decision-making models by integrating Aczél-Alsina aggregation with bipolar neutrosophic theory, offering a novel approach to handling ambiguity and conflicting information.

## 1. Introduction

Effective inventory management is essential for preserving operational excellence, raising customer happiness, and optimizing profitability in the fast-paced retail industry. Managing inventory throughout its network of 50 stores posed substantial issues for RetailPlus, a growing retail chain specializing in consumer electronics. The business suffered from inaccurate inventory records, ineffective replenishment procedures, low real-time visibility, excessive carrying costs, and a decline in customer satisfaction due to stockouts and overstocking.

RetailPlus started a revolutionary journey to restructure its inventory management procedures to address these issues. The organization used various cutting-edge digital solutions to optimize workflows, augment data precision, and elevate overall efficacy. This case study examines the major digital innovations that RetailPlus implemented, emphasizing their data-driven replenishment strategy, sophisticated inventory management system, improvements to real-time visibility, and initiatives to reduce upfront costs. RetailPlus sought to address its inventory management problems and create a more responsive, economical, and customer-focused business by utilizing cutting-edge technologies and data-driven approaches. Due to the enormous differences in needs between traditional and internet retail, this change presents significant operational issues. These variations include demand drivers, product diversity, ideal inventory configurations, cost structures, supply chain setups, and delivery techniques [1]. We recommend that four capability organizations brand management, merchandising, service, and promote data management are shaped by distinct unions of a foundation [2]. Lessees and lessors of national tenants typically employ a risk-sharing retail leasing model, which combines a fixed rental with a risk-sharing element known as a turnover clause [3]. Due to issues with keeping the right amount of inventory on hand, RetailPlus frequently finds itself in a situation where some of its most popular items are overstocked and require precious resources [4]. Many problems, such as incorrect orders, misplaced items, and inaccurate stock levels, can result from erroneous inventory data. Inaccuracies in recorded vs actual inventory levels have caused problems for RetailPlus regarding order fulfillment and ineffective stock management [5]. Using this model, two alternative deployment strategies are examined, which are defined by the decisions made about order allocation, inventory positioning, delivery schema, and inbound flow patterns. The advanced placement of inventory at a fulfillment canter in the city where online orders are requested is also considered in the second deployment plan [6].

Therefore, numerous scholars have proposed various innovative concepts to tackle these common challenges. In 1965, Zadeh [7] introduced fuzzy sets, diverging from the principles of traditional crisp logic by acknowledging that properties possess a certain degree of vagueness. Utilizing fuzziness allows for more precise handling of numerous complex real-life problems that are difficult to describe in crisp terms. The foundation of the fuzzy set model is the membership grade, which extends the traditional crisp model by measuring an element’s level of membership to a set. Values for membership fall between 0 and 1, with values nearer 1 denoting a higher degree of membership. To handle a variety of ambiguous problems, academics have consequently developed several sophisticated variations of fuzzy sets, such as intuitionistic fuzzy sets [8]. Some properties of the proposed similarity measures were discussed, and the related similarity measures were also compared [9]. Tahir et al. [10] proposed the inaugurated DM framework to prioritize the numerous types of computer vision by considering artificial data. Tian et al. [11] exposed the improved linguistic interval-valued Atanassov intuitionistic fuzzy weighted averaging (ILIVAIFWA) AO of LIVAIFNs. Xu et al. [12] proposed several hesitant-ordered weighted distance measures and hesitant-ordered weighted similarity measures. Smarandache et al. [13] proposed the neutrosophic set. Smarandache et al. [14] proposed the defines the neutrosophic set. One gives examples from mathematics, physics, philosophy, and applications of the neutrosophic set. Wang et al. [15] proposed the single-valued neutrosophic set. Wang et al. [16] proposed the interval neutrosophic set. Zhang et al. [17] proposed the interval neutrosophic sets (INSs), proposed exactly to address issues with a set of numbers in the real unit interval. Zhang et al. [18] proposed a weighted correlation coefficient measure of INSs, a decision-making method. Huang [19] proposed the similarity measure, the entropy measure, and the index of distance. Shahzadi et al. [20] proposed the single-valued neutrosophic set. Broumi et al. [21] proposed the concept of correlation coefficients of interval-valued neutrosophic set (INS for short). Chi et al. [22] proposed the TOPSIS to INS, and concerning the multiple attribute decisdecision-makinglems. Deli et al. [23] proposed the score, certainty, and accuracy functions to compare the bipolar neutrosophic sets. Ulucay et al. [24] proposed the multi-criteria decision-making method for bipolar neutrosophic set.

### 1.1. Background and related work

Ali et al. [25] proposed the Aczel-Alsina t-norm (AATNRM) and Aczel-Alsina t-conorm (AATCRM). Senapati et al. [26] proposed the HF Aczel–Alsina hybrid geometric (HFAAHG) operator and HF Aczel–Alsina weighted Bonferroni mean (HFAAWBM) operator. Senapati et al. [27] proposed the MADM technique dependent on the advanced IVIF aggregation operators. Senapati et al. [28] created some IF aggregation operators, for example, the IF Aczel–Alsina weighted averaging operator, the IF Aczel–Alsina ordered weighted averaging operator, and the IF Aczel–Alsina hybrid averaging operator. To validate the validity of the myths that are derived from them, the writers completed an example of a MADM technique [29]. For the MADM process, an algorithm and pseudocode were created that are intended to handle unpredictable events involving BFNs in real life [30]. A decision-making algorithm is created and used for two intricate multi-polar site selection problems based on the suggested AOs [31]. Utilizing similarity measurements, the inferior ratio method was expanded for the C-IFS framework [32]. The inferior ratio strategy, which is reliant on similarity metrics, was extended for the C-IFS framework [33]. The Archimedean Heronian aggregation operator in complex intuitionistic fuzzy form is demonstrated [34]. Fahmi et al. [35] demonstrated the solution to complicated real-life problems, an algorithm for the MADM problem. Fahmi et al. [36] proposed the focus on parameter different solutions of Fuzzy TOPSIS Positive ideal and Negative ideal solutions for efficient decision making. Fahmi et al. [37] proposed new methods based on developed aggregation and geometric operators. Fahmi et al. [38] proposed the TFF-AHP-TOPSIS technique deliberated and a PIS and NIS. Fahmi et al. [39] proposed various properties of these operators and derived the relationship between the proposed operators and the exiting aggregation operators. Fahmi et al. [40] proposed the extend the classical VIKOR method to solve the MCDM method based on triangular cubic fuzzy numbers. Fahmi et al. [41] proposed the various properties of these operators and derive the relationship between the proposed operators and the exiting aggregation operators. For more details one can see [48–50].

Senapati et al. [42] introduced the SW triangular norm-based approach that aggregates group preferences, facilitating a systematic decision-making process. Triangular norms ensure a realistic representation of interrelationships among decision criteria, leading to optimal healthcare supply chain management solutions. Kakati et al. [43] depicted the feasibility of the proposed MADM method, a thorough quantitative model. Siab et al. [44] proposed the linguistic interval-valued intuitionistic fuzzy Aczel-Alsina hybrid weighted averaging (LIVIFAAHWA) operator and linguistic interval-valued intuitionistic fuzzy Aczel-Alsina hybrid weighted geometric (LIVIFAAHWG) operators. Senapati [45] proposed the SVN AA order weighted average (SVNAAOWA) operator and SVN AA hybrid average (SVNAAHA) operator. Imran et al. [46] proposed a Multi-Attribute Decision-Making (MADM) method for the proposed operators under the consideration of CSVN information. Ali et al. [47] introduced the best one among the above four, we illustrate the technique of multi-attribute decision-making (MADM) procedure based on initiated operators to show the supremacy and validity of the proposed theory.

Several researchers have explored extensions of fuzzy and neutrosophic sets to improve their ability to handle uncertainty. Studies on generalized neutrosophic sets and their applications in decision-making and computational intelligence have laid the groundwork for our proposed GBNS. For example, recent works on multi-valued neutrosophic models have demonstrated the benefits of extending conventional frameworks with more adaptable membership functions. Additionally, research in bipolar information systems highlights the need for models that simultaneously manage positive and negative aspects of data. Our approach builds on these foundational concepts by integrating bipolarity with generalized membership structures, offering a more powerful and versatile tool for uncertainty modeling. This theoretical advancement addresses practical challenges in decision-making environments, where the complexity of information often exceeds the expressive capacity of standard models. Furthermore, the proposed GBNS framework enhances computational intelligence applications by improving decision-making accuracy and resilience in the presence of conflicting and incomplete information.

### 1.2. Novelty

The novelty of this research lies in introducing a comprehensive framework for Generalized Bipolar Neutrosophic Sets (GBNS) with several significant theoretical and practical advancements:

1. Generalized Bipolar Neutrosophic Numbers (GBNNs): An Introduction
  By defining GBNNs, we expand the current Bipolar Neutrosophic Set and allow for a more versatile representation of contradictory, ambiguous, and uncertain data. This invention offers a more comprehensive and flexible mathematical framework, addressing the drawbacks of conventional Bipolar Neutrosophic models.
2. Novel Operational Laws for GBNNs: We create new, logically sound operational laws to facilitate intricate GBNN-based mathematical procedures. When working with data from the real world, these rules guarantee precision, closure, and computational efficiency.
3. enhanced Score, Accuracy, and Certainty Functions: To improve ranking and decision-making in the face of ambiguity, we provide enhanced score and accuracy functions. In situations involving ambiguous and insufficient data, the new functions enable more accurate comparisons, leading to better decision outcomes.
4. Creation of Advanced Aggregation Operators: We provide six cutting-edge aggregation operators for GBNNs that are intended to resolve conflicts and evaluate dependability while integrating data from various sources. These operators offer a more thorough and flexible method of combining ambiguous data.
5. Proposal of a Multi-Criteria Decision-Making (MCDM) Model:
  We design a robust MCDM framework based on the proposed GBNN operators and evaluation functions. This method effectively handles complex decision-making problems by supporting dynamic evaluations in uncertain and multi-criteria environments.
6. Application and numerical validation:
  To demonstrate the effectiveness of our proposed framework, we present a detailed numerical example, highlighting its practical application in real-world problems such as expert evaluation, risk assessment, and strategic decision-making.

### 1.2. Contribution of the paper

This investigation aims to devise a strategic and insightful technique for recommending the most suitable alternative from a set of options. We have formulated AA t-NMs and t-CNMs with BN aggregation operators. The primary focus of the concepts of GBNAAWA, GBNAAOWA, GBNAAHWA, GBNAAWG, GBNAAOWG, and GBNAAHWG operators within the BNS framework. Furthermore, we demonstrate the effectiveness of diverse AOs. Ultimately, the paper achieves the following key milestones:

1. To introduce new AOs like the GBNAAWA, GBNAAOWA, GBNAAHWA, GBNAAWG, GBNAAOWG, and GBNAAHWG within the BNS framework, it is essential to explore the fundamental operations of t-NMs and t-CNMs.
2. Explore the innovative operators and provide specific examples of their applications.
3. Develop an algorithm that addresses multiple attribute decision-making issues using BN data.
4. Discuss the BN data to assess the reliability and utility of the proposed method.
5. Conduct a comparative analysis contrasting existing AOs with our proposed ones, summarizing these comparisons to comprehensively demonstrate the suggested AOs’ effectiveness.
6. Perform sensitivity analyses to demonstrate the reliability and robustness of the proposed method.

### 1.3. Motivation

In real-world decision-making and information processing, uncertainty, inconsistency, and conflicting opinions frequently arise. Various mathematical frameworks, including fuzzy sets, intuitionistic fuzzy sets, and neutrosophic sets, have been developed to address these issues by considering multiple dimensions of information. Among them, the Bipolar Neutrosophic Set (BNS) stands out due to its ability to represent positive and negative information simultaneously through degrees of truth, indeterminacy, and falsity. This unique capability makes BNS a valuable tool for modeling complex and contradictory data in domains such as multi-criteria decision-making, medical diagnosis, and sentiment analysis.

Despite its usefulness, the conventional BNS framework has notable limitations due to its fixed structure. It assumes static membership functions, which restrict its ability to handle complex, dynamic, and context-dependent uncertain data. Real-world applications often demand a more adaptable and expressive representation to capture subtle variations in information.

To overcome these constraints, we propose the Generalized Bipolar Neutrosophic Set (GBNS), which extends the traditional BNS by introducing a more flexible membership structure. GBNS supports generalized and adaptive membership functions, enabling more precise and context-aware modeling of uncertain, vague, and incomplete information. This enhancement provides a comprehensive mathematical framework capable of accommodating a broader range of real-life problems where traditional models fall short.

The manuscript is structured as follows: In Section 2, we provide a concise overview of bipolar neutrosophic sets and aggregation operators. Section 3 introduces six novel aggregation operators based on Aczel-Alsina operations within the BNS framework, exploring their desirable properties. Section 4 applies these operators to define a Group Decision Making issue. A case study on interpreting construction project decisions using bipolar neutrosophic techniques is presented to demonstrate their practicality in section 5. The section 6 is presented in the conclusion.

A list of abbreviations in Table 1 is given as

**Table 1:** A list of abbreviations.

Abbreviations	Full Name
BNNs	Bipolar neutrosophic numbers
MCDM	Multi-criteria decision making
MADM	Multi-Attribute Decision Making
BNAA	Bipolar neutrosophic Aczel-Alsina
GBNS	Generalized bipolar neutrosophic sets
GBNAAWA	Generalized Bipolar neutrosophic Aczel-Alsina weighted averaging
GBNAAOWA	Generalized Bipolar neutrosophic Aczel-Alsina ordered weighted averaging
GBNAAHWA	Generalized Bipolar neutrosophic Aczel-Alsina hybrid weighted averaging
GBNAAWG	Generalized Bipolar neutrosophic Aczel-Alsina weighted geometric
GBNAAOWG	Generalized Bipolar neutrosophic Aczel-Alsina ordered weighted geometric
GBNAAHWG	Generalized Bipolar neutrosophic Aczel-Alsina weighted hybrid geometric
ILIVAIFWA	improved linguistic interval-valued Atanassov intuitionistic fuzzy weighted averaging
LIVAIFNs	linguistic interval-valued Atanassov intuitionistic fuzzy numbers
AO	Aggregation operators
INSs	interval neutrosophic sets
AATNRM	Aczel-Alsina t-norm
AATCRM	Aczel-Alsina t-conorm
HFAAHG	Hesitant Fuzzy Aczel–Alsina hybrid geometric operator
HFAAWBM	Hesitant Fuzzy Aczel–Alsina weighted Bonferroni mean
IF	Intuitionistic fuzzy
SVNAAOWA	single-valued neutrosophic Aczel-Alsina order weighted average
SVNAAHA	single-valued neutrosophic Aczel-Alsina hybrid weighted average
CSVN	Complex Single-Valued Neutrosophic
PIS	Positive ideal solution
NIS	Negative ideal solution
fuzzy setintuitionisticCircular	C-IFS

## 2. Preliminaries

We developed the basic definition and properties.

**Definition.2.1.**[7] *Let* Φ≠Z *and by a fuzzy set* $\gamma =\left\{\left\langle x,\mu_{\gamma (y)}\right\rangle \;|\;y\in Z\right\},$ *in which* $\mu_{\gamma (x)}$ *is a mapping from* Z *to* [0,1] *present the membership grade of an element* y *in* Z.

**Definition.2.2.**[17] *Let* Z *be a universal set, A neutrosophic set* N *in* Z *is define as* $A=\left\{\left\langle v,\Gamma_{A(v)},\Omega_{A\;(v)},\Upsilon_{A\;(v)}\;|\;v\in Z\right\rangle \right\}$ *where* $\Gamma_{A\;(v)},$ $\Omega_{A\;(v)},$ $\Upsilon_{A\;(v)}$ *are the truth membership function and the indeterminacy function and the falsity membership function respectively, such that* Γ,Ω,Υ,: $X\to ]0^{-}\;,1^{+}[$ *and* $0^{-}\leq \Gamma_{A\;(v)}+\Omega_{A\;(v)}+\Upsilon_{A\;(v)}\leq 3^{+}.$

**Definition.2.3.**[20] *Let* Z *be a universal set and let an element* y∈Z*. An interval-valued neutrosophic set* T *in* Z *is* T. $=\left\{y,\;\left(A_{A}(y),\;B_{A}(y),\;C_{A}(y),\right)\right\},$ *where* $A_{A}(y),$ $B_{A}(y),$ $C_{A}(y),$ *are the truth-membership, indeterminacy-membership, and falsity membership functions separately, for each point* y *in* Z *we have that* 0≤ Sup $A_{A}(y)+Sup$ $B_{A}(y)+Sup$ $C_{A}(y)\leq 3.$

### 2.1. BNNs

In this subsection, we define the definition and score function.

**Definition.2.1.1.** [19] *A bipolar neutrosophic number* A *in* M *is define as* $A=\{\begin{array}{c} \left(\begin{array}{c} v,\vartheta^{+}(v)\\ P^{+}(v)\\ Q^{+}(v)\\ \vartheta^{-}(v)\\ P^{-}(v)\\ Q^{-}(v), \end{array}\right)\\ :\;\;v\in M \end{array}\},$ *where* $\vartheta^{+},P^{+},Q^{+}\;\;:\;\;M$ →[0,1] *and* $\;\;\vartheta^{-},P^{-},Q^{-}\;\;:\;\;M$ →[−1,0]. *The positive membership degree* $\vartheta^{+}(v),P^{+}(v),Q^{+}(v)$ *denotes the truth membership, in terminate membership and false membership of an element* v∈M *corresponding to the bipolar set* A *and the negative membership degree* $\vartheta^{-}(v),P^{-}(v),Q^{-}(v)$ *denotes the truth membership, interminate membership, and false membership of an element* v∈M *to some implicit counter-property corresponding to a bipolar set* A.

**Definition.2.1.2.**[29] *Assume* $P=\left\{\begin{array}{c} \left[\begin{array}{c} {\text{\textbackslash{}DH}}^{+},\\ {\text{\textbackslash{}AE}}^{+},\\ {\text{\textbackslash{}OE}}^{+} \end{array}\right],\\ \left[\begin{array}{c} {\text{\textbackslash{}DH}}^{-},\\ {\text{\textbackslash{}AE}}^{-},\\ {\text{\textbackslash{}OE}}^{-} \end{array}\right] \end{array}\right\}$ *The score function* S(P), *accuracy function* H(P) *and certainty function* Q(P) *of a bipolar neutrosophic number are defined as follows:*


$$S(P)=\frac{1}{6}\left({\text{\textbackslash{}DH}}^{+}+1-{\text{\textbackslash{}AE}}^{+}+1-{\text{\textbackslash{}OE}}^{+}+1+{\text{\textbackslash{}DH}}^{-}-{\text{\textbackslash{}AE}}^{-}-{\text{\textbackslash{}OE}}^{-}\right)$$

### 2.2. Operational laws of Aczel-Alsina

In this subsection, we define the operational laws and related preparties.

**Definition.2.2.1**. *Assume* $P_{1}=\{\begin{array}{c} {}[{\text{\textbackslash{}DH}}_{1}^{+}\\ {\text{\textbackslash{}AE}}_{1}^{+}\\ {\text{\textbackslash{}OE}}_{1}^{+}],\\ {}[{\text{\textbackslash{}DH}}_{1}^{-}\\ {\text{\textbackslash{}AE}}_{1}^{-}\\ {\text{\textbackslash{}OE}}_{1}] \end{array}\}$ *and* $P_{2}=\{\begin{array}{c} {\text{\textbackslash{}DH}}_{2}^{+}\\ {\text{\textbackslash{}AE}}_{2}^{+}\\ {\text{\textbackslash{}OE}}_{2}^{+}\\ {\text{\textbackslash{}DH}}_{2}^{-}\\ {\text{\textbackslash{}AE}}_{2}^{-}\\ {\text{\textbackslash{}OE}}_{2}^{-} \end{array}\}$ *consist of two bipolar neutrosophic number. Then the define*


(P)

$$P_{1}⊕P_{2}=\{\begin{array}{c} {}[1-e^{-{\left(-In(1-{\text{\textbackslash{}DH}}_{1}^{+}){)}^{\Lambda }+(-In(1-{\text{\textbackslash{}DH}}_{2}^{+}){)}^{\Lambda }\right)}^{\frac{1}{\Lambda }}},\\ 1-e^{-{\left(-In{\text{\textbackslash{}AE}}_{1}^{+}{)}^{\Lambda }+(-In{\text{\textbackslash{}AE}}_{2}^{+}{)}^{\Lambda }\right)}^{\frac{1}{\Lambda }}},\\ 1-e^{-{\left(-In{\text{\textbackslash{}OE}}_{1}^{+}{)}^{\Lambda }+(-In{\text{\textbackslash{}OE}}_{2}^{+}{)}^{\Lambda }\right)}^{\frac{1}{\Lambda }}}]\\ {}[-(1-e^{-{\left(-In{\text{\textbackslash{}DH}}_{1}^{-}{)}^{\Lambda }+(-In{\text{\textbackslash{}DH}}_{2}^{-}{)}^{\Lambda }\right)}^{\frac{1}{\Lambda }}}),\\ -(1-e^{-{\left(-In(1-{\text{\textbackslash{}AE}}_{1}^{-}){)}^{\Lambda }+(-In(1-{\text{\textbackslash{}AE}}_{2}^{-}){)}^{\Lambda }\right)}^{\frac{1}{\Lambda }}}),\\ -(1-e^{-{\left(-In(1-{\text{\textbackslash{}OE}}_{1}^{-}){)}^{\Lambda }+(-In(1-{\text{\textbackslash{}OE}}_{2}^{-}){)}^{\Lambda }\right)}^{\frac{1}{\Lambda }}})] \end{array}\};$$

(b)

$$P_{1}⊗P_{2}=\{\begin{array}{c} {}[1-e^{-{\left(-In{\text{\textbackslash{}DH}}_{1}^{+}{)}^{\Lambda }+(-In{\text{\textbackslash{}DH}}_{2}^{+}{)}^{\Lambda }\right)}^{\frac{1}{\Lambda }}},\\ 1-e^{-{\left(-In(1-{\text{\textbackslash{}AE}}_{1}^{+}){)}^{\Lambda }+(-In(1-{\text{\textbackslash{}AE}}_{2}^{+}){)}^{\Lambda }\right)}^{\frac{1}{\Lambda }}},\\ 1-e^{-{\left(-In(1-{\text{\textbackslash{}OE}}_{1}^{+}){)}^{\Lambda }+(-In(1-{\text{\textbackslash{}OE}}_{2}^{+}){)}^{\Lambda }\right)}^{\frac{1}{\Lambda }}}],\\ {}[-(1-e^{-{\left(-In(1-{\text{\textbackslash{}DH}}_{1}^{-}){)}^{\Lambda }+(-In(1-{\text{\textbackslash{}DH}}_{2}^{-}){)}^{\Lambda }\right)}^{\frac{1}{\Lambda }}}),\\ -(1-e^{-{\left(-In{\text{\textbackslash{}AE}}_{1}^{-}{)}^{\Lambda }+(-In{\text{\textbackslash{}AE}}_{2}^{-}{)}^{\Lambda }\right)}^{\frac{1}{\Lambda }}}),\\ -(1-e^{-{\left(-In{\text{\textbackslash{}OE}}_{1}^{-}{)}^{\Lambda }+(-In{\text{\textbackslash{}OE}}_{2}^{-}{)}^{\Lambda }\right)}^{\frac{1}{\Lambda }}})] \end{array}\};$$

(c)

$$\lambda P_{1}=\{\begin{array}{c} {}[1-e^{-{\left(\lambda (-In(1-{\text{\textbackslash{}DH}}_{1}^{+}){)}^{\Lambda }\right)}^{\frac{1}{\Lambda }}},\\ e^{-{\left(\lambda (-In{\text{\textbackslash{}AE}}_{1}^{+}{)}^{\Lambda }\right)}^{\frac{1}{\Lambda }}},\\ e^{-{\left(\lambda (-In{\text{\textbackslash{}OE}}_{1}^{+}{)}^{\Lambda }\right)}^{\frac{1}{\Lambda }}}]\\ {}[-e^{-{\left(\lambda (-In{\text{\textbackslash{}DH}}_{1}^{-}{)}^{\Lambda }\right)}^{\frac{1}{\Lambda }}},\\ -(1-e^{-{\left(\lambda (-In(1-{\text{\textbackslash{}AE}}_{1}^{-}){)}^{\Lambda }\right)}^{\frac{1}{\Lambda }}}),\\ -(1-e^{-{\left(\lambda (-In(1-{\text{\textbackslash{}OE}}_{1}^{-}){)}^{\Lambda }\right)}^{\frac{1}{\Lambda }}}) \end{array}\},$$

(d)

$$P_{1}^{\lambda }=\{\begin{array}{c} {}[e^{-{\left(\lambda (-In{\text{\textbackslash{}DH}}_{1}^{+}{)}^{\Lambda }\right)}^{\frac{1}{\Lambda }}},\\ 1-e^{-{\left(\lambda (-In(1-{\text{\textbackslash{}AE}}_{1}^{+}){)}^{\Lambda }\right)}^{\frac{1}{\Lambda }}},\\ 1-e^{-{\left(\lambda (-In(1-{\text{\textbackslash{}OE}}_{1}^{+}){)}^{\Lambda }\right)}^{\frac{1}{\Lambda }}}],\\ {}[-(1-e^{-{\left(\lambda (-In(1-{\text{\textbackslash{}DH}}_{1}^{-}){)}^{\Lambda }\right)}^{\frac{1}{\Lambda }}}),\\ -e^{-{\left(\lambda (-In{\text{\textbackslash{}AE}}_{1}^{-}{)}^{\Lambda }\right)}^{\frac{1}{\Lambda }}},\\ -e^{-{\left(\lambda (-In{\text{\textbackslash{}OE}}_{1}^{-}{)}^{\Lambda }\right)}^{\frac{1}{\Lambda }}}] \end{array}\}.$$

**Theorem.2.2.2.** *Let* $Y=\{\begin{array}{c} {\text{\textbackslash{}DH}}^{+}\\ {\text{\textbackslash{}AE}}^{+}\\ {\text{\textbackslash{}OE}}^{+}\\ {\text{\textbackslash{}DH}}^{-}\\ {\text{\textbackslash{}AE}}^{-}\\ {\text{\textbackslash{}OE}}^{-} \end{array}\},$ $Y_{1}=\{\begin{array}{c} {\text{\textbackslash{}DH}}_{1}^{+}\\ {\text{\textbackslash{}AE}}_{1}^{+}\\ {\text{\textbackslash{}OE}}_{1}^{+}\\ {\text{\textbackslash{}DH}}_{1}^{-}\\ {\text{\textbackslash{}AE}}_{1}^{-}\\ {\text{\textbackslash{}OE}}_{1}^{-} \end{array}\}$ *and* $Y_{2}=\{\begin{array}{c} {\text{\textbackslash{}DH}}_{2}^{+}\\ {\text{\textbackslash{}AE}}_{2}^{+}\\ {\text{\textbackslash{}OE}}_{2}^{+}\\ {\text{\textbackslash{}DH}}_{2}^{-}\\ {\text{\textbackslash{}AE}}_{2}^{-}\\ {\text{\textbackslash{}OE}}_{2}^{-} \end{array}\}$ *be three BNAANs and* $\lambda ,\lambda_{1}\lambda_{2}>0,$ *then we have*

(1)
$Y_{2}⊕Y_{1}=Y_{1}⊕Y_{2};$

(2)
$Y_{2}⊗Y_{1}=Y_{1}⊗Y_{2};$

(3)
$\lambda (Y_{1}⊕Y_{2})=\lambda Y_{1}⊕\lambda Y_{2};$

(4)
$\lambda (Y_{1}⊗Y_{2})=\lambda Y_{1}⊗\lambda Y_{2};$

(5)
$\lambda_{1}Y⊕\lambda_{2}Y=Y(\lambda_{1}Y⊕\lambda_{2});$

(6)
$Y(\lambda_{1}⊗\lambda_{2})=\lambda_{1}Y⊗\lambda_{2}Y;$

(7)
$(Y_{1}⊗Y_{2}{)}^{\lambda }=Y_{1}^{\lambda }⊗Y_{2}^{\lambda };$

(8)
$(Y_{1}⊕Y_{2}{)}^{\lambda }=Y_{1}^{\lambda }⊕Y_{2}^{\lambda };$

(9)
$Y^{\lambda_{1}}⊗Y^{\lambda_{2}}=Y^{\lambda_{1}⊕\lambda_{2}}.$

## 3. Bipolar neutrosophic based on Aczel-Alsina aggregation operators

In this section, we define six aggregation operators.

### 3.1. GBNAAWA operator

**Definition.3.1.1.** *Assume* $L=\left\{\begin{array}{c} \left[\begin{array}{c} U_{L}^{+},\\ {\text{\textbackslash{}AE}}_{L}^{+},\\ {\text{\textbackslash{}OE}}_{L}^{+} \end{array}\right],\\ \left[\begin{array}{c} U_{L}^{-},\\ {\text{\textbackslash{}AE}}_{L}^{-},\\ {\text{\textbackslash{}OE}}_{L}^{-} \end{array}\right] \end{array}\right\}$ *be a family of BNN, it is referred to as a GBNAAWA operator if it satisfies:*


$$\text{GBNAAWL}(L_{1},L_{2},...,L_{m}{)}^{T}=R⊕\left(\overset{m}{\underset{Q=1}{⊕}}\lambda_{Q}L_{Q}\right)$$

*And* $\lambda_{Q}$ *is the weight vector* ${\;L}_{V}(V=1,2,\ldots m)$ $\lambda_{V}\in [0,1]$ *and* $\sum_{V=1}^{m}\lambda_{V}=1.$

**Theorem. 3.1.2.** *Let* $L=\left\{\begin{array}{c} \left[\begin{array}{c} U_{L}^{+},\\ {\text{\textbackslash{}AE}}_{L}^{+},\\ {\text{\textbackslash{}OE}}_{L}^{+} \end{array}\right],\\ \left[\begin{array}{c} U_{L}^{-},\\ {\text{\textbackslash{}AE}}_{L}^{-},\\ {\text{\textbackslash{}OE}}_{L}^{-} \end{array}\right] \end{array}\right\}$ *represent a family of bipolar neutrosophic numbers. It is termed as the GBNAAWA operator if it satisfies:GBNAAWA* $\left(L_{1},L_{2},...,L_{m}{)}^{T}=\{\begin{array}{c} {}[1-e^{-{\left(\sum_{P=1}^{m}\lambda (U_{P}^{+}\left(-Im(1-U_{P}^{+}){)}^{\Lambda }\right)\right)}^{\frac{1}{\Lambda }}},\\ e^{-{\left(\sum_{P=1}^{m}\lambda ({\text{\textbackslash{}AE}}_{P}^{+}\left(-Im{\text{\textbackslash{}AE}}_{P}^{+}\right){)}^{\Lambda }\right)}^{\frac{1}{\Lambda }}},\\ e^{-{\left(\sum_{P=1}^{m}\lambda \left({\text{\textbackslash{}OE}}_{P}^{+}(-Im{\text{\textbackslash{}OE}}_{P}^{+}{)}^{\Lambda }\right)\right)}^{\frac{1}{\Lambda }}}]\\ {}[-e^{-{\left(\sum_{P=1}^{m}\lambda \left(U_{P}^{-}(-ImU_{P}^{-}{)}^{\Lambda }\right)\right)}^{\frac{1}{\Lambda }}},\\ -(1-e^{-{\left(\sum_{P=1}^{m}\lambda \left({\text{\textbackslash{}AE}}_{P}^{-}(-Im(1-{\text{\textbackslash{}AE}}_{P}^{-}){)}^{\Lambda }\right)\right)}^{\frac{1}{\Lambda }}}),\\ -(1-e^{-{\left(\sum_{P=1}^{m}\lambda \left({\text{\textbackslash{}OE}}_{P}^{-}(-Im(1-{\text{\textbackslash{}OE}}_{P}^{-}){)}^{\Lambda }\right)\right)}^{\frac{1}{\Lambda }}}) \end{array}\right\}$

*where* $\lambda_{P}$ *is the weight of* $L_{V}(V=1,2,\ldots m)$ $\lambda_{V}\in [0,1]$ *and* $\sum_{V=1}^{m}\lambda_{V}=1.$

Theorem. 3.1.3. ***(Idempotency):****If* $L=\left\{\begin{array}{c} \left[\begin{array}{c} U_{L}^{+},\\ {\text{\textbackslash{}AE}}_{L}^{+},\\ {\text{\textbackslash{}OE}}_{L}^{+} \end{array}\right],\\ \left[\begin{array}{c} U_{L}^{-},\\ {\text{\textbackslash{}AE}}_{L}^{-},\\ {\text{\textbackslash{}OE}}_{L}^{-} \end{array}\right] \end{array}\right\}$ *for all* P=1,2,3...,m, *then GBNAAWA* $(L_{1},L_{2},...,L_{m})=L.$

Theorem. 3.1.4. ***(Commutativity):****If* $\left(L_{1,}^{\prime }L_{2}^{\prime },...,L_{m}^{\prime }\right)$ *is any permutation of* $\left(L_{1,}L_{2},...,L_{m}\right)$*, then GBNAAWA* $(L_{1,}^{\prime }L_{2}^{\prime },...,L_{m}^{\prime })=$ *GBNAAWA* $\left(L_{1,}L_{2},...,L_{m}\right)$

Theorem. 3.1.5. ***(Boundedness):****If* $S^{-}=min(L_{1},L_{2},...,L_{m}),$ $S^{+}$ = $\max(L_{1},L_{2},...,L_{m}),$ *then* $S^{-}\leq$ *GBNAAWA* $(L_{1,}L_{2},...,L_{m})\leq$ $S^{+}.$

### 3.2. GBNAAOWA operator

**Definition.3.2.1.** *Let* $L=\left\{\begin{array}{c} \left[\begin{array}{c} U_{L}^{+},\\ {\text{\textbackslash{}AE}}_{L}^{+},\\ {\text{\textbackslash{}OE}}_{L}^{+} \end{array}\right],\\ \left[\begin{array}{c} U_{L}^{-},\\ {\text{\textbackslash{}AE}}_{L}^{-},\\ {\text{\textbackslash{}OE}}_{L}^{-} \end{array}\right] \end{array}\right\}$ *represent a family of bipolar neutrosophic numbers. It is termed as the GBNAAOWA operator if it satisfies:*


$$\text{GBNAAOWL}(L_{1},L_{2},...,L_{m}{)}^{T}=R⊕\left(\overset{m}{\underset{P=1}{⊕}}\lambda_{P}L_{P}\right)$$

*And* $\lambda_{P}$ *is the weight of* $L_{V}(V=1,2,\ldots m)$ $\lambda_{V}\in [0,1]$ *and* $\sum_{V=1}^{m}\lambda_{V}=1.$

**Theorem. 3.2.2.** *Let* $L=\left\{\begin{array}{c} \left[\begin{array}{c} U_{L}^{+},\\ {\text{\textbackslash{}AE}}_{L}^{+},\\ {\text{\textbackslash{}OE}}_{L}^{+} \end{array}\right],\\ \left[\begin{array}{c} U_{L}^{-},\\ {\text{\textbackslash{}AE}}_{L}^{-},\\ {\text{\textbackslash{}OE}}_{L}^{-} \end{array}\right] \end{array}\right\}$ *represent a family of bipolar neutrosophic numbers. It is termed as the GBNAAOWA operator if it satisfies:GBNAAOWA* $(L_{1},L_{2},...,L_{m}{)}^{T}=$ $\left\{\begin{array}{c} {}[1-e^{-{\left(\sum_{P=1}^{m}\lambda (U_{P}^{+}\left(-Im(1-U_{P}^{+}){)}^{\Lambda }\right)\right)}^{\frac{1}{\Lambda }}},\\ e^{-{\left(\sum_{P=1}^{m}\lambda ({\text{\textbackslash{}AE}}_{P}^{+}\left(-Im{\text{\textbackslash{}AE}}_{P}^{+}\right){)}^{\Lambda }\right)}^{\frac{1}{\Lambda }}},\\ e^{-{\left(\sum_{P=1}^{m}\lambda \left({\text{\textbackslash{}OE}}_{P}^{+}(-Im{\text{\textbackslash{}OE}}_{P}^{+}{)}^{\Lambda }\right)\right)}^{\frac{1}{\Lambda }}}]\\ {}[-e^{-{\left(\sum_{P=1}^{m}\lambda \left(U_{P}^{-}(-ImU_{P}^{-}{)}^{\Lambda }\right)\right)}^{\frac{1}{\Lambda }}},\\ -(1-e^{-{\left(\sum_{P=1}^{m}\lambda \left({\text{\textbackslash{}AE}}_{P}^{-}(-Im(1-{\text{\textbackslash{}AE}}_{P}^{-}){)}^{\Lambda }\right)\right)}^{\frac{1}{\Lambda }}}),\\ -(1-e^{-{\left(\sum_{P=1}^{m}\lambda \left({\text{\textbackslash{}OE}}_{P}^{-}(-Im(1-{\text{\textbackslash{}OE}}_{P}^{-}){)}^{\Lambda }\right)\right)}^{\frac{1}{\Lambda }}}) \end{array}\right\}$

*where* $\lambda_{P}$ *is the weight of* $L_{V}(V=1,2,\ldots m)$ $\lambda_{V}\in [0,1]$ *and* $\sum_{V=1}^{m}\lambda_{V}=1.$

**Theorem. 3.2.3. *(Idempotency):****If* $L=\left\{\begin{array}{c} \left[\begin{array}{c} U_{L}^{+},\\ {\text{\textbackslash{}AE}}_{L}^{+},\\ {\text{\textbackslash{}OE}}_{L}^{+} \end{array}\right],\\ \left[\begin{array}{c} U_{L}^{-},\\ {\text{\textbackslash{}AE}}_{L}^{-},\\ {\text{\textbackslash{}OE}}_{L}^{-} \end{array}\right] \end{array}\right\}$ *for all* Q=1,2,3...,m, *then GBNAAOWA* $(L_{1},L_{2},...,L_{m})=L.$

**Theorem. 3.2.4. *(Commutativity):****If* $\left(L_{1,}^{\prime }L_{2}^{\prime },...,L_{m}^{\prime }\right)$ *is any permutation of* $\left(L_{1,}L_{2},...,L_{m}\right)$*, then GBNAAOWA* $(L_{1,}^{\prime }L_{2}^{\prime },...,L_{m}^{\prime })=$ *GBNAAOWA* $\left(L_{1,}L_{2},...,L_{m}\right)$

**Theorem. 3.2.5. *(Boundedness):****If* $S^{-}=min(L_{1},L_{2},...,L_{n}),$ $S^{+}$ = $\max(L_{1},L_{2},...,L_{n}),$ *then* $S^{-}\leq$ *GBNAAOWA* $(L_{1,}L_{2},...,L_{n})\leq$ $S^{+}.$

### 3.3. GBNAAHWA operator

**Definition. 3.3.1.** *Let* $L=\left\{\begin{array}{c} \left[\begin{array}{c} U_{L}^{+},\\ {\text{\textbackslash{}AE}}_{L}^{+},\\ {\text{\textbackslash{}OE}}_{L}^{+} \end{array}\right],\\ \left[\begin{array}{c} U_{L}^{-},\\ {\text{\textbackslash{}AE}}_{L}^{-},\\ {\text{\textbackslash{}OE}}_{L}^{-} \end{array}\right] \end{array}\right\}$ *represent a family of bipolar neutrosophic numbers. It is termed as the GBNAAHWA operator if it satisfies:*


$$\text{GBNAAHWA}(L_{1},L_{2},...,L_{m}{)}^{T}=R⊕\left(\overset{m}{\underset{Q=1}{⊕}}\lambda_{Q}L_{Q}\right)$$

*And* $\lambda_{Q}$ *is the weight of* $L_{V}(V=1,2,\ldots m)$ $\lambda_{V}\in [0,1]$ *and* $\sum_{V=1}^{m}\lambda_{V}=1.$

**Theorem. 3.3.2.** *Let* $L=\left\{\begin{array}{c} \left[\begin{array}{c} U_{L}^{+},\\ {\text{\textbackslash{}AE}}_{L}^{+},\\ {\text{\textbackslash{}OE}}_{L}^{+} \end{array}\right],\\ \left[\begin{array}{c} U_{L}^{-},\\ {\text{\textbackslash{}AE}}_{L}^{-},\\ {\text{\textbackslash{}OE}}_{L}^{-} \end{array}\right] \end{array}\right\}$ *represent a family of bipolar neutrosophic numbers. It is termed as the GBNAAHWA operator if it satisfies GBNAAWG* $(L_{1},L_{2},...,L_{m}{)}^{T}=$

: $\left\{\begin{array}{c} {}[1-e^{-{\left(\sum_{P=1}^{m}\lambda (U_{P}^{+}\left(-Im(1-U_{P}^{+}){)}^{\Lambda }\right)\right)}^{\frac{1}{\Lambda }}},\\ e^{-{\left(\sum_{P=1}^{m}\lambda ({\text{\textbackslash{}AE}}_{P}^{+}\left(-Im{\text{\textbackslash{}AE}}_{P}^{+}\right){)}^{\Lambda }\right)}^{\frac{1}{\Lambda }}},\\ e^{-{\left(\sum_{P=1}^{m}\lambda \left({\text{\textbackslash{}OE}}_{P}^{+}(-Im{\text{\textbackslash{}OE}}_{P}^{+}{)}^{\Lambda }\right)\right)}^{\frac{1}{\Lambda }}}]\\ {}[-e^{-{\left(\sum_{P=1}^{m}\lambda \left(U_{P}^{-}(-ImU_{P}^{-}{)}^{\Lambda }\right)\right)}^{\frac{1}{\Lambda }}},\\ -(1-e^{-{\left(\sum_{P=1}^{m}\lambda \left({\text{\textbackslash{}AE}}_{P}^{-}(-Im(1-{\text{\textbackslash{}AE}}_{P}^{-}){)}^{\Lambda }\right)\right)}^{\frac{1}{\Lambda }}}),\\ -(1-e^{-{\left(\sum_{P=1}^{m}\lambda \left({\text{\textbackslash{}OE}}_{P}^{-}(-Im(1-{\text{\textbackslash{}OE}}_{P}^{-}){)}^{\Lambda }\right)\right)}^{\frac{1}{\Lambda }}}) \end{array}\right\}$

*where* $\lambda_{P}$ *is the weight of* $L_{V}(V=1,2,\ldots m)$ $\lambda_{V}\in [0,1]$ *and* $\sum_{V=1}^{m}\lambda_{V}=1.$

**Theorem. 3.3.3. *(Idempotency):****If* $L=\left\{\begin{array}{c} \left[\begin{array}{c} U_{L}^{+},\\ {\text{\textbackslash{}AE}}_{L}^{+},\\ {\text{\textbackslash{}OE}}_{L}^{+} \end{array}\right],\\ \left[\begin{array}{c} U_{L}^{-},\\ {\text{\textbackslash{}AE}}_{L}^{-},\\ {\text{\textbackslash{}OE}}_{L}^{-} \end{array}\right] \end{array}\right\}$ *for all* V=1,2,3...,m, *then GBNAAHWA* $(L_{1},L_{2},...,L_{n})=L.$

**Theorem. 3.3.4. *(Commutativity):****If* $\left(L_{1,}^{\prime }L_{2}^{\prime },...,L_{n}^{\prime }\right)$ *is any permutation of* $\left(L_{1,}L_{2},...,L_{m}\right)$*, then GBNAAHWA* $(L_{1,}^{\prime }L_{2}^{\prime },...,L_{m}^{\prime })=$ *GBNAAHWA* $\left(L_{1,}L_{2},...,L_{m}\right)$

**Theorem. 3.3.5. *(Boundedness):****If* $S^{-}=min(L_{1},L_{2},...,L_{m}),$ $S^{+}=$ $\max(L_{1},L_{2},...,L_{m}),$ *then* $S^{-}\leq$ *GBNAAHWA* $(L_{1,}L_{2},...,L_{m})\leq$ $S^{+}.$

### 3.4. GBNAAWG operator

**Definition.3.4.1.** *Let* $L=\left\{\begin{array}{c} \left[\begin{array}{c} U_{L}^{+},\\ {\text{\textbackslash{}AE}}_{L}^{+},\\ {\text{\textbackslash{}OE}}_{L}^{+} \end{array}\right],\\ \left[\begin{array}{c} U_{L}^{-},\\ {\text{\textbackslash{}AE}}_{L}^{-},\\ {\text{\textbackslash{}OE}}_{L}^{-} \end{array}\right] \end{array}\right\}$ *represent a family of bipolar neutrosophic numbers. It is termed as the GBNAAWG operator if it satisfies: GBNAAWG* $(L_{1},L_{2},...,L_{m}{)}^{T}=R⊗\left({⊗}_{V=1}^{m}L_{V}^{\lambda }\right),$ *where* $\lambda_{P}$ *is the weight of* $L_{V}(V=1,2,\ldots m)$ $\lambda_{V}\in [0,1]$ *and* $\sum_{V=1}^{m}\lambda_{V}=1.$

**Theorem. 3.4.2.** *Let* $L=\left\{\begin{array}{c} \left[\begin{array}{c} U_{L}^{+},\\ {\text{\textbackslash{}AE}}_{L}^{+},\\ {\text{\textbackslash{}OE}}_{L}^{+} \end{array}\right],\\ \left[\begin{array}{c} U_{L}^{-},\\ {\text{\textbackslash{}AE}}_{L}^{-},\\ {\text{\textbackslash{}OE}}_{L}^{-} \end{array}\right] \end{array}\right\}$ *represent a family of bipolar neutrosophic numbers. It is termed as the GBNAAWG operator if it satisfies: GBNAAOWG* $\left(L_{1},L_{2},...,L_{m}{)}^{T}=\{\begin{array}{c} {}[e^{-{\left(\sum_{V=1}^{m}\lambda (U_{L}^{+}(-ImU_{L}^{+}){)}^{\Lambda }\right)}^{\frac{1}{\Lambda }}},\\ 1-e^{-{\left(\sum_{V=1}^{m}\lambda ({\text{\textbackslash{}AE}}_{L}^{+}(-Im(1-{\text{\textbackslash{}AE}}_{L}^{+})){)}^{\Lambda }\right)}^{\frac{1}{\Lambda }}},\\ 1-e^{-{\left(\sum_{V=1}^{m}\lambda ({\text{\textbackslash{}OE}}_{L}^{+}(-Im(1-{\text{\textbackslash{}OE}}_{L}^{+})){)}^{\Lambda }\right)}^{\frac{1}{\Lambda }}}],\\ {}[-(1-e^{-{\left(\sum_{V=1}^{m}\lambda (U_{L}^{-}(-Im(1-U_{L}^{-})){)}^{\Lambda }\right)}^{\frac{1}{\Lambda }}}),\\ -e^{-{\left(\sum_{V=1}^{m}\lambda ({\text{\textbackslash{}AE}}_{L}^{-}(-Im{\text{\textbackslash{}AE}}_{L}^{-}{)}^{\Lambda })\right)}^{\frac{1}{\Lambda }}},\\ -e^{-{\left(\sum_{V=1}^{m}\lambda ({\text{\textbackslash{}OE}}_{L}^{-}(-Im{\text{\textbackslash{}OE}}_{L}^{-}{)}^{\Lambda })\right)}^{\frac{1}{\Lambda }}}] \end{array}\right\}$

*where* $\lambda_{V}$ *is the weight of* $L_{V}(V=1,2,\ldots m)$ $\lambda_{V}\in [0,1]$ *and* $\sum_{V=1}^{m}\lambda_{V}=1.$

**Theorem. 3.4.3. *(Idempotency):****If* $L=\left\{\begin{array}{c} \left[\begin{array}{c} U_{L}^{+},\\ {\text{\textbackslash{}AE}}_{L}^{+},\\ {\text{\textbackslash{}OE}}_{L}^{+} \end{array}\right],\\ \left[\begin{array}{c} U_{L}^{-},\\ {\text{\textbackslash{}AE}}_{L}^{-},\\ {\text{\textbackslash{}OE}}_{L}^{-} \end{array}\right] \end{array}\right\}$ *for all* V=1,2,3...,m, *then GBNAAWG* $(L_{1},L_{2},...,L_{m})=L.$

**Theorem. 3.4.4. *(Commutativity):****If* $\left(Q_{1,}^{\prime }Q_{2}^{\prime },...,Q_{m}^{\prime }\right)$ *is any permutation of* $\left(Q_{1,}Q_{2},...,Q_{m}\right)$*, then GBNAAWG* $(Q_{1,}^{\prime }Q_{2}^{\prime },...,Q_{m}^{\prime })=$ *GBNAAWG* $\left(Q_{1,}Q_{2},...,Q_{m}\right)$

**Theorem. 3.4.5. *(Boundedness):****If* $S^{-}=min(M_{1},M_{2},...,M_{n}),$ $S^{+}$ = $\max(M_{1},M_{2},...,M_{n}),$ *then* $S^{-}\leq$ *GBNAAWG* $(M_{1,}M_{2},...,M_{n})\leq$ $S^{+}.$

### 3.5.GBNAAOWG operator

**Definition.3.5.1.** *Let* $L=\left\{\begin{array}{c} \left[\begin{array}{c} U_{L}^{+},\\ {\text{\textbackslash{}AE}}_{L}^{+},\\ {\text{\textbackslash{}OE}}_{L}^{+} \end{array}\right],\\ \left[\begin{array}{c} U_{L}^{-},\\ {\text{\textbackslash{}AE}}_{L}^{-},\\ {\text{\textbackslash{}OE}}_{L}^{-} \end{array}\right] \end{array}\right\}$ *represent a family of bipolar neutrosophic numbers. It is termed as the GBNAAOWG operator if it satisfies: GBNAAOWG* $(Q_{1},Q_{2},...,Q_{m}{)}^{T}=R⊗\left({⊗}_{V=1}^{m}L_{V}^{\lambda }\right),$ *where* $\lambda_{V}$ *is the weight of* $L_{V}(V=1,2,\ldots m)$ $\lambda_{V}\in [0,1]$ *and* $\sum_{V=1}^{m}\lambda_{V}=1.$

**Theorem. 3.5.2.** *Let* $L=\left\{\begin{array}{c} \left[\begin{array}{c} U_{L}^{+},\\ {\text{\textbackslash{}AE}}_{L}^{+},\\ {\text{\textbackslash{}OE}}_{L}^{+} \end{array}\right],\\ \left[\begin{array}{c} U_{L}^{-},\\ {\text{\textbackslash{}AE}}_{L}^{-},\\ {\text{\textbackslash{}OE}}_{L}^{-} \end{array}\right] \end{array}\right\}$ *represent a family of bipolar neutrosophic numbers. It is termed as the GBNAAOWG operator if it satisfies:*

*GBNAAOWG*


$$\left(L_{1},L_{2},...,L_{m}{)}^{T}=\{\begin{array}{c} {}[e^{-{\left(\sum_{V=1}^{m}\lambda (U_{L}^{+}(-ImU_{L}^{+}){)}^{\Lambda }\right)}^{\frac{1}{\Lambda }}},\\ 1-e^{-{\left(\sum_{V=1}^{m}\lambda ({\text{\textbackslash{}AE}}_{L}^{+}(-Im(1-{\text{\textbackslash{}AE}}_{L}^{+})){)}^{\Lambda }\right)}^{\frac{1}{\Lambda }}},\\ 1-e^{-{\left(\sum_{V=1}^{m}\lambda ({\text{\textbackslash{}OE}}_{L}^{+}(-Im(1-{\text{\textbackslash{}OE}}_{L}^{+})){)}^{\Lambda }\right)}^{\frac{1}{\Lambda }}}],\\ {}[-(1-e^{-{\left(\sum_{V=1}^{m}\lambda (U_{L}^{-}(-Im(1-U_{L}^{-})){)}^{\Lambda }\right)}^{\frac{1}{\Lambda }}}),\\ -e^{-{\left(\sum_{V=1}^{m}\lambda ({\text{\textbackslash{}AE}}_{L}^{-}(-Im{\text{\textbackslash{}AE}}_{L}^{-}{)}^{\Lambda })\right)}^{\frac{1}{\Lambda }}},\\ -e^{-{\left(\sum_{V=1}^{m}\lambda ({\text{\textbackslash{}OE}}_{L}^{-}(-Im{\text{\textbackslash{}OE}}_{L}^{-}{)}^{\Lambda })\right)}^{\frac{1}{\Lambda }}}] \end{array}\right\}$$

*where* $\lambda_{V}$ *is the weight of* $L_{V}(V=1,2,\ldots m)$ $\lambda_{V}\in [0,1]$ *and* $\sum_{V=1}^{m}\lambda_{V}=1.$

**Theorem. 3.5.3. *(Idempotency):****If* $L=\left\{\begin{array}{c} \left[\begin{array}{c} U_{L}^{+},\\ {\text{\textbackslash{}AE}}_{L}^{+},\\ {\text{\textbackslash{}OE}}_{L}^{+} \end{array}\right],\\ \left[\begin{array}{c} U_{L}^{-},\\ {\text{\textbackslash{}AE}}_{L}^{-},\\ {\text{\textbackslash{}OE}}_{L}^{-} \end{array}\right] \end{array}\right\}$ *for all* P=1,2,3...,n, *then GBNAAOWG* $(L_{1},L_{2},...,L_{n})=L.$

**Theorem, 3.5.4. *(Commutativity):****If* $\left(Q_{1,}^{\prime }Q_{2}^{\prime },...,Q_{n}^{\prime }\right)$ *is any permutation of* $\left(Q_{1,}Q_{2},...,Q_{n}\right)$*, then GBNAAOWG* $(Q_{1,}^{\prime }Q_{2}^{\prime },...,Q_{n}^{\prime })=$ *GBNAAOWG* $\left(Q_{1,}Q_{2},...,Q_{n}\right)$

**Theorem. 3.5.5. *(Boundedness):****If* $\;S^{-}=min(W_{1},W_{2},...,W_{n}),$ $S^{+}$ = $\max(W_{1},W_{2},...,W_{n}),$ *then* $S^{-}\leq$ *GBNAAOWG* $(W_{1,}W_{2},...,W_{n})\leq$ $S^{+}.$

### 3.6. GBNAAHWG operator

**Definition.3.6.1.** *Let* $Q=\left\{\begin{array}{c} \left[\begin{array}{c} U_{Q}^{+},\\ {\text{\textbackslash{}AE}}_{Q}^{+},\\ {\text{\textbackslash{}OE}}_{Q}^{+} \end{array}\right],\\ \left[\begin{array}{c} U_{Q}^{-},\\ {\text{\textbackslash{}AE}}_{Q}^{-},\\ {\text{\textbackslash{}OE}}_{Q}^{-} \end{array}\right] \end{array}\right\}$ *represent a family of bipolar neutrosophic numbers. It is termed as the GBNAAHWG operator if it satisfies: GBNAAHWG* $(Q_{1},Q_{2},...,Q_{m}{)}^{T}=R⊗\left({⊗}_{V=1}^{n}Q_{P}^{\lambda }\right),$ *where* $\lambda_{V}$ *is the weight of* $Q_{V}(V=1,2,\ldots m)$ $\lambda_{V}\in [0,1]$ *and* $\sum_{V=1}^{m}\lambda_{V}=1.$

**Theorem.3.6.2.** *Let* $L=\left\{\begin{array}{c} \left[\begin{array}{c} U_{L}^{+},\\ {\text{\textbackslash{}AE}}_{L}^{+},\\ {\text{\textbackslash{}OE}}_{L}^{+} \end{array}\right],\\ \left[\begin{array}{c} U_{L}^{-},\\ {\text{\textbackslash{}AE}}_{L}^{-},\\ {\text{\textbackslash{}OE}}_{L}^{-} \end{array}\right] \end{array}\right\}$ *represent a family of bipolar neutrosophic numbers. It is termed as the GBNAAHWG operator if it satisfies:*

*GBNAAHWG*


$$\left(L_{1},L_{2},...,L_{m}{)}^{T}=\{\begin{array}{c} {}[e^{-{\left(\sum_{V=1}^{m}\lambda (U_{L}^{+}(-ImU_{L}^{+}){)}^{\Lambda }\right)}^{\frac{1}{\Lambda }}},\\ 1-e^{-{\left(\sum_{V=1}^{m}\lambda ({\text{\textbackslash{}AE}}_{L}^{+}(-Im(1-{\text{\textbackslash{}AE}}_{L}^{+})){)}^{\Lambda }\right)}^{\frac{1}{\Lambda }}},\\ 1-e^{-{\left(\sum_{V=1}^{m}\lambda ({\text{\textbackslash{}OE}}_{L}^{+}(-Im(1-{\text{\textbackslash{}OE}}_{L}^{+})){)}^{\Lambda }\right)}^{\frac{1}{\Lambda }}}],\\ {}[-(1-e^{-{\left(\sum_{V=1}^{m}\lambda (U_{L}^{-}(-Im(1-U_{L}^{-})){)}^{\Lambda }\right)}^{\frac{1}{\Lambda }}}),\\ -e^{-{\left(\sum_{V=1}^{m}\lambda ({\text{\textbackslash{}AE}}_{L}^{-}(-Im{\text{\textbackslash{}AE}}_{L}^{-}{)}^{\Lambda })\right)}^{\frac{1}{\Lambda }}},\\ -e^{-{\left(\sum_{V=1}^{m}\lambda ({\text{\textbackslash{}OE}}_{L}^{-}(-Im{\text{\textbackslash{}OE}}_{L}^{-}{)}^{\Lambda })\right)}^{\frac{1}{\Lambda }}}] \end{array}\right\}$$

*where* $\lambda_{V}$ *is the weight of* $L_{V}(V=1,2,\ldots m)$ $\lambda_{V}\in [0,1]$ *and* $\sum_{V=1}^{m}\lambda_{V}=1.$

**Theorem. 3.6.3. *(Idempotency):****If* $L=\left\{\begin{array}{c} \left[\begin{array}{c} U_{L}^{+},\\ {\text{\textbackslash{}AE}}_{L}^{+},\\ {\text{\textbackslash{}OE}}_{L}^{+} \end{array}\right],\\ \left[\begin{array}{c} U_{L}^{-},\\ {\text{\textbackslash{}AE}}_{L}^{-},\\ {\text{\textbackslash{}OE}}_{L}^{-} \end{array}\right] \end{array}\right\}$ *for all* V=1,2,3...,m, *then GBNAAHWG* $(L_{1},L_{2},...,L_{m})=L.$

**Theorem. 3.6.4. *(Commutativity):****If* $\left(Q_{1,}^{\prime }Q_{2}^{\prime },...,Q_{n}^{\prime }\right)$ *is any permutation of* $\left(Q_{1,}Q_{2},...,Q_{n}\right)$*, then GBNAAHWG* $(Q_{1,}^{\prime }Q_{2}^{\prime },...,Q_{n}^{\prime })=$ *GBNAAHWG* $\left(Q_{1,}Q_{2},...,Q_{n}\right)$

**Theorem. 3.6.5. *(Boundedness):****If* $S^{-}=min(G_{1},G_{2},...,G_{m}),$ $S^{+}$ = $\max(G_{1},G_{2},...,G_{m}),$ *then* $S^{-}\leq$ *GBNAAHWG* $(G_{1,}G_{2},...,G_{m})\leq$ $S^{+}.$

## 4. MCDM based on BNN

In this part, we define a proposed operator-based decision-making strategy for the MCDM issue in the context of the BN.

$A_{1,}A_{2,}....A_{m}$ among the m choices, and n attributes $G_{1,}G_{2},...G_{n}$ have weight vectors $w_{\text{\textbackslash{}DH}}$ =1,2,..n such that $w_{\text{\textbackslash{}DH}}>0$ and $\sum_{j=1}^{n}w_{j}=1,$ take into a GDM issue. Assume that $\lambda =(\lambda_{1},\lambda_{2},..\lambda_{\text{\textbackslash{}DH}})$ present the decision-makers and $w=(w_{1},w_{2},...,w_{n}{)}^{\text{\textbackslash{}DH}}$. Suppose that the alternatives $A_{k}(k=1,2,..m)$ over the attributes $G_{\text{\textbackslash{}DH}}(=1,2,..n)$ is evaluated by decision-maker $\lambda_{\text{\textbackslash{}DH}}(=1,2,..,n)$ and offer the preference in the form of BNNs $\alpha =(\begin{array}{c} {\text{\textbackslash{}DH}}^{+}\\ {\text{\textbackslash{}AE}}^{+}\\ {\text{\textbackslash{}OE}}^{+}\\ {\text{\textbackslash{}DH}}^{-}\\ {\text{\textbackslash{}AE}}^{-}\\ {\text{\textbackslash{}OE}}^{-} \end{array})$.

To explain the DM strategy based on the operation,

the BN decision matrix must be calculated in Step 1.

Step 2: Describe the operator for GBNAAWA.

GBNAAWA


$$\left(L_{1},L_{2},...,L_{n}{)}^{T}=\{\begin{array}{c} {}[1-e^{-{\left(\sum_{P=1}^{n}\lambda (U_{P}^{+}\left(-In(1-U_{P}^{+}){)}^{\Lambda }\right)\right)}^{\frac{1}{\Lambda }}},\\ e^{-{\left(\sum_{P=1}^{n}\lambda ({\text{\textbackslash{}AE}}_{P}^{+}\left(-In{\text{\textbackslash{}AE}}_{P}^{+}\right){)}^{\Lambda }\right)}^{\frac{1}{\Lambda }}},\\ e^{-{\left(\sum_{P=1}^{n}\lambda \left({\text{\textbackslash{}OE}}_{P}^{+}(-In{\text{\textbackslash{}OE}}_{P}^{+}{)}^{\Lambda }\right)\right)}^{\frac{1}{\Lambda }}}]\\ {}[-e^{-{\left(\sum_{P=1}^{n}\lambda \left(U_{P}^{-}(-InU_{P}^{-}{)}^{\Lambda }\right)\right)}^{\frac{1}{\Lambda }}},\\ -(1-e^{-{\left(\sum_{P=1}^{n}\lambda \left({\text{\textbackslash{}AE}}_{P}^{-}(-In(1-{\text{\textbackslash{}AE}}_{P}^{-}){)}^{\Lambda }\right)\right)}^{\frac{1}{\Lambda }}}),\\ -(1-e^{-{\left(\sum_{P=1}^{n}\lambda \left({\text{\textbackslash{}OE}}_{P}^{-}(-In(1-{\text{\textbackslash{}OE}}_{P}^{-}){)}^{\Lambda }\right)\right)}^{\frac{1}{\Lambda }}}) \end{array}\right\}$$

Step 3 Define the GBNAAWA operator

GBNAAWA


$$\left(L_{1},L_{2},...,L_{n}{)}^{T}=\{\begin{array}{c} {}[1-e^{-{\left(\sum_{P=1}^{n}\lambda (U_{P}^{+}\left(-In(1-U_{P}^{+}){)}^{\Lambda }\right)\right)}^{\frac{1}{\Lambda }}},\\ e^{-{\left(\sum_{P=1}^{n}\lambda ({\text{\textbackslash{}AE}}_{P}^{+}\left(-In{\text{\textbackslash{}AE}}_{P}^{+}\right){)}^{\Lambda }\right)}^{\frac{1}{\Lambda }}},\\ e^{-{\left(\sum_{P=1}^{n}\lambda \left({\text{\textbackslash{}OE}}_{P}^{+}(-In{\text{\textbackslash{}OE}}_{P}^{+}{)}^{\Lambda }\right)\right)}^{\frac{1}{\Lambda }}}]\\ {}[-e^{-{\left(\sum_{P=1}^{n}\lambda \left(U_{P}^{-}(-InU_{P}^{-}{)}^{\Lambda }\right)\right)}^{\frac{1}{\Lambda }}},\\ -(1-e^{-{\left(\sum_{P=1}^{n}\lambda \left({\text{\textbackslash{}AE}}_{P}^{-}(-In(1-{\text{\textbackslash{}AE}}_{P}^{-}){)}^{\Lambda }\right)\right)}^{\frac{1}{\Lambda }}}),\\ -(1-e^{-{\left(\sum_{P=1}^{n}\lambda \left({\text{\textbackslash{}OE}}_{P}^{-}(-In(1-{\text{\textbackslash{}OE}}_{P}^{-}){)}^{\Lambda }\right)\right)}^{\frac{1}{\Lambda }}}) \end{array}\right\}$$

Step 4 Computing the score function


$$S(a)=\frac{1}{6}\left({\text{\textbackslash{}DH}}^{+}+1-{\text{\textbackslash{}AE}}^{+}+1-{\text{\textbackslash{}OE}}^{+}+1+{\text{\textbackslash{}DH}}^{-}-{\text{\textbackslash{}AE}}^{-}-{\text{\textbackslash{}OE}}^{-}\right)$$

Step 5: Find the ranking.

## 5. Case study

With 50 locations in several states, RetailPlus is a mid-sized retail chain that specializes in consumer electronics. Even with its expansion and market presence, RetailPlus has encountered many difficulties with inventory control. These difficulties have had an effect on overall profitability, customer satisfaction, and operational efficiency.

**Issue Description**

The real and recorded inventory levels frequently differed at RetailPlus. These errors resulted in stockouts of some items and overstocks of others, which upset customers and hurt sales.

**Ineffective Resupply Procedures**

Resupply was done manually and reactively. Rather than using current data, store managers made requests for inventory replenishment based more on intuition or past sales data.

**Absence of Real-Time Perception**

Across its network of outlets, RetailPlus lacked real-time visibility into inventory levels. The inventory management system was antiquated and did not allow system integrations or real-time data updates.

**Excessive Carrying Expenses**

Excessive inventory meant high carrying expenses for the company. Among the expenses were storage, insurance, and possible obsolescence of unsold goods.

**Unhappiness with Customers**

Customer discontent was caused by inconsistent inventory levels since they regularly encountered out-of-stock items or couldn’t find requested products.

### 5.1. Illustrative example


$$DIG_{1}\;\;:$$

Putting in Place a Sophisticated Inventory Management System 

A state-of-the-art inventory management system with real-time tracking features was implemented by RetailPlus. The updated system included features like:

Real-time data synchronization made it possible to update inventory levels in real-time across all retail locations and distribution hubs.

Automated replenishment: Resupply procedures were automated using data-driven algorithms in accordance with sales projections and inventory levels.

Connectivity with Different Systems: integrated to offer a single picture of inventory with supply chain management tools and point-of-sale (POS) systems.


$$DIG_{2}\;\;:$$

Data-Informed Resupply Approach 

The business changed its replenishment strategy from being reactive to proactive:

Sales Forecasting: Made use of sophisticated analytics to predict demand and modify stock levels in accordance.

Inventory Optimization: Applied optimization methods to minimize stockouts, reduce surplus inventory, and balance inventory levels.


$$DIG_{3}\;\;:$$

Improving Visibility in Real Time 

RetailPlus enhanced its inventory visibility in real time by implementing:

Centralized Dashboard: To track inventory levels, sales patterns, and replenishment requirements, a centralized dashboard was created and distributed to store managers and the central office.

Mobile Access: Store managers may now access inventory data on their phones and use it to make quick, well-informed choices.


$$DIG_{4}\;\;:$$

Lowering Upfront Expenses 

Reduce carrying costs by using RetailPlus:

Streamlined Inventory: aimed at cutting back on excess inventory by using just-in-time inventory procedures and improved forecasting.

Optimized warehouse: By combining inventories and lowering the number of storage facilities, warehouse efficiency was increased.

Table 2 is given as

**Table 2:** Values.

DIG	Attribute	Category
$DIG_{1}$	Real-Time Data Synchronization	System Integration
$DIG_{1}$	Automated Replenishment	Process Automation
$DIG_{1}$	Connectivity with Different Systems	System Integration
$DIG_{2}$	Sales Forecasting	Predictive Analytics
$DIG_{2}$	Inventory Optimization	Inventory Management
$DIG_{3}$	Centralized Dashboard	Data Visualization
$DIG_{3}$	Mobile Access	Accessibility and Mobility
$DIG_{4}$	Streamlined Inventory	Cost Management
$DIG_{4}$	Optimized Warehouse	Operational Efficiency

The weight vector i s (0.2,0.1,0.3,0.4). Tables 3 and 4 display the built bipolar neutrosophic decision matrices.

**Table 3:** Decision bipolar neutrosophic disorder.

	$DIG_{1}$	$DIG_{2}$	$DIG_{3}$	$DIG_{4}$
$VV_{1}$	$\left\{\begin{array}{c} {}[0.09,\\ 0.23,\\ 0.26],\\ {}[-0.03,\\ -0.20,\\ -0.41] \end{array}\right\}$	$\left\{\begin{array}{c} {}[0.02,\\ 0.13,\\ 0.18],\\ {}[-0.17,\\ -0.47,\\ -0.48] \end{array}\right\}$	$\left\{\begin{array}{c} {}[0.17,\\ 0.19,\\ 0.21],\\ {}[-0.04,\\ -0.16,\\ -0.18] \end{array}\right\}$	$\left\{\begin{array}{c} {}[0.21,\\ 0.23,\\ 0.29],\\ {}[-0.07,\\ -0.09,\\ -0.11] \end{array}\right\}$
$VV_{2}$	$\left\{\begin{array}{c} {}[0.17,\\ 0.19,\\ 0.21],\\ {}[-0.04,\\ -0.16,\\ -0.18] \end{array}\right\}$	$\left\{\begin{array}{c} {}[0.09,\\ 0.23,\\ 0.26,\\ {}[-0.03,\\ -0.20,\\ -0.41] \end{array}\right\}$	$\left\{\begin{array}{c} {}[0.02,\\ 0.13,\\ 0.18],\\ {}[-0.17,\\ -0.47,\\ -0.48] \end{array}\right\}$	$\left\{\begin{array}{c} {}[0.07,\\ 0.13,\\ 0.18],\\ {}[-0.03,\\ -0.07,\\ -0.11] \end{array}\right\}$
$VV_{3}$	$\left\{\begin{array}{c} {}[0.02,\\ 0.13,\\ 0.18],\\ {}[-0.17,\\ -0.47,\\ -0.48] \end{array}\right\}$	$\left\{\begin{array}{c} {}[0.21,\\ 0.23,\\ 0.29],\\ {}[-0.07,\\ -0.09,\\ -0.11] \end{array}\right\}$	$\left\{\begin{array}{c} {}[0.09,\\ 0.23,\\ 0.26,\\ {}[-0.03,\\ -0.20,\\ -0.41] \end{array}\right\}$	$\left\{\begin{array}{c} {}[0.17,\\ 0.19,\\ 0.21],\\ {}[-0.04,\\ -0.16,\\ -0.18] \end{array}\right\}$
$VV_{4}$	$\left\{\begin{array}{c} {}[0.21,\\ 0.23,\\ 0.29],\\ {}[-0.07,\\ -0.09,\\ -0.11] \end{array}\right\}$	$\left\{\begin{array}{c} {}[0.02,\\ 0.13,\\ 0.18],\\ {}[-0.17,\\ -0.47,\\ -0.48] \end{array}\right\}$	$\left\{\begin{array}{c} {}[0.17,\\ 0.19,\\ 0.21],\\ {}[-0.04,\\ -0.16,\\ -0.18] \end{array}\right\}$	$\left\{\begin{array}{c} {}[0.09,\\ 0.23,\\ 0.26,\\ {}[-0.03,\\ -0.20,\\ -0.41] \end{array}\right\}$

**Table 4:** Bipolar neutrosophic decision Table 4.

	$DIG_{1}$	$DIG_{2}$	$DIG_{3}$	$DIG_{4}$
$VV_{1}$	$\left\{\begin{array}{c} {}[0.01,\\ 0.2,\\ 0.3,\\ {}[-0.02,\\ -0.11,\\ -0.13] \end{array}\right\}$	$\left\{\begin{array}{c} {}[0.07,\\ 0.13,\\ 0.18],\\ {}[-0.03,\\ -0.07,\\ -0.11] \end{array}\right\}$	$\left\{\begin{array}{c} {}[0.17,\\ 0.19,\\ 0.21],\\ {}[-0.04,\\ -0.16,\\ -0.18] \end{array}\right\}$	$\left\{\begin{array}{c} {}[0.1,\\ 0.3,\\ 0.9],\\ {}[-0.02,\\ -0.04,\\ -0.09] \end{array}\right\}$
$VV_{2}$	$\left\{\begin{array}{c} {}[0.17,\\ 0.19,\\ 0.21],\\ {}[-0.04,\\ -0.16,\\ -0.18] \end{array}\right\}$	$\left\{\begin{array}{c} {}[0.01,\\ 0.2,\\ 0.3,\\ {}[-0.02,\\ -0.11,\\ -0.13] \end{array}\right\}$	$\left\{\begin{array}{c} {}[0.1,\\ 0.3,\\ 0.9],\\ {}[-0.02,\\ -0.04,\\ -0.09] \end{array}\right\}$	$\left\{\begin{array}{c} {}[0.03,\\ 0.11,\\ 0.14],\\ {}[-0.03,\\ -0.07,\\ -0.11] \end{array}\right\}$
$VV_{3}$	$\left\{\begin{array}{c} {}[0.1,\\ 0.3,\\ 0.9],\\ {}[-0.02,\\ -0.04,\\ -0.09] \end{array}\right\}$	$\left\{\begin{array}{c} {}[0.07,\\ 0.13,\\ 0.18],\\ {}[-0.03,\\ -0.07,\\ -0.11] \end{array}\right\}$	$\left\{\begin{array}{c} {}[0.01,\\ 0.2,\\ 0.3,\\ {}[-0.02,\\ -0.11,\\ -0.13] \end{array}\right\}$	$\left\{\begin{array}{c} {}[0.17,\\ 0.19,\\ 0.21],\\ {}[-0.04,\\ -0.16,\\ -0.18] \end{array}\right\}$
$VV_{4}$	$\left\{\begin{array}{c} {}[0.07,\\ 0.13,\\ 0.18],\\ {}[-0.03,\\ -0.07,\\ -0.11] \end{array}\right\}$	$\left\{\begin{array}{c} {}[0.02,\\ 0.13,\\ 0.18],\\ {}[-0.17,\\ -0.47,\\ -0.48] \end{array}\right\}$	$\left\{\begin{array}{c} {}[0.17,\\ 0.19,\\ 0.21],\\ {}[-0.04,\\ -0.16,\\ -0.18] \end{array}\right\}$	$\left\{\begin{array}{c} {}[0.01,\\ 0.2,\\ 0.3,\\ {}[-0.02,\\ -0.11,\\ -0.13] \end{array}\right\}$

The bipolar neutrosophic fuzzy decision Tables 3 and 4 must be calculated in step 1.

Step 2 Exhibit the GBNAAWA operator using weights w=(0.3,0.2,0.4,0.1), as shown in Table 5

**Table 5:** GBNAAWA operator.

	$DIG_{1}$	$DIG_{2}$	$DIG_{3}$	$DIG_{4}$
$VV_{1}$	$\left\{\begin{array}{c} {}[0.2098,\\ 0.2345,\\ 0.2654],\\ {}[-0.0369,\\ -0.2036,\\ -0.4145] \end{array}\right\}$	$\left\{\begin{array}{c} {}[0.1078,\\ 0.1369,\\ 0.1874],\\ {}[-0.1789,\\ -0.4712,\\ -0.4878] \end{array}\right\}$	$\left\{\begin{array}{c} {}[0.1745,\\ 0.1982,\\ 0.2123],\\ {}[-0.0456,\\ -0.1642,\\ -0.1896] \end{array}\right\}$	$\left\{\begin{array}{c} {}[0.2123,\\ 0.2896,\\ 0.2854],\\ {}[-0.0345,\\ -0.2785,\\ -0.4562] \end{array}\right\}$
$VV_{2}$	$\left\{\begin{array}{c} {}[0.1789,\\ 0.1896,\\ 0.1783],\\ {}[-0.1778,\\ -0.4963,\\ -0.4789] \end{array}\right\}$	$\left\{\begin{array}{c} {}[0.2098,\\ 0.2345,\\ 0.2654],\\ {}[-0.0369,\\ -0.2036,\\ -0.4145] \end{array}\right\}$	$\left\{\begin{array}{c} {}[0.1103,\\ 0.1104,\\ 0.1105],\\ {}[-0.1101,\\ -0.1203,\\ -0.1004] \end{array}\right\}$	$\left\{\begin{array}{c} {}[0.1078,\\ 0.1369,\\ 0.1874],\\ {}[-0.1789,\\ -0.4712,\\ -0.4878] \end{array}\right\}$
$VV_{3}$	$\left\{\begin{array}{c} {}[0.1745,\\ 0.1982,\\ 0.2123],\\ {}[-0.0456,\\ -0.1642,\\ -0.1896] \end{array}\right\}$	$\left\{\begin{array}{c} {}[0.1078,\\ 0.1369,\\ 0.1874],\\ {}[-0.1789,\\ -0.4712,\\ -0.4878] \end{array}\right\}$	$\left\{\begin{array}{c} {}[0.2098,\\ 0.2345,\\ 0.2654],\\ {}[-0.0369,\\ -0.2036,\\ -0.4145] \end{array}\right\}$	$\left\{\begin{array}{c} {}[0.0236,\\ 0.0459,\\ 0.1125],\\ {}[-0.1456,\\ -0.1698,\\ -0.1896] \end{array}\right\}$
$VV_{4}$	$\left\{\begin{array}{c} {}[0.1745,\\ 0.1982,\\ 0.2123],\\ {}[-0.0456,\\ -0.1642,\\ -0.1896] \end{array}\right\}$	$\left\{\begin{array}{c} {}[0.1078,\\ 0.1369,\\ 0.1874],\\ {}[-0.1789,\\ -0.4712,\\ -0.4878] \end{array}\right\}$	$\left\{\begin{array}{c} {}[0.2098,\\ 0.2345,\\ 0.2654],\\ {}[-0.0369,\\ -0.2036,\\ -0.4145] \end{array}\right\}$	$\left\{\begin{array}{c} {}[0.0236,\\ 0.0459,\\ 0.1125],\\ {}[-0.1456,\\ -0.1698,\\ -0.1896] \end{array}\right\}$

Step 3:Define the GBNAAWA operator w=(0.3,0.2,0.4,0.1) and Table 6 is given as

**Table 6:** GBNAAWA operator.

$VV_{1}$	$\left\{\begin{array}{c} {}[0.3117,0.4524,\\ 0.6825],[-0.2987,\\ -0.2023,-0.2867] \end{array}\right\}$
$VV_{2}$	$\left\{\begin{array}{c} {}[0.2003,0.2804,\\ 0.2905],[-0.2271,\\ -0.4123,-0.4559] \end{array}\right\}$
$VV_{3}$	$\left\{\begin{array}{c} {}[0.5303,0.5404,\\ 0.5975],[-0.6631,\\ -0.7213,-0.8244] \end{array}\right\}$
$VV_{4}$	$\left\{\begin{array}{c} {}[0.5098,0.5876,\\ 0.7609],[-0.0981,\\ -0.7654,-0.8987] \end{array}\right\}$

Step 4: Determine the score function.


$$f_{1}=0.0122,f_{2}=0.6834,f_{3}=0.1006,f_{4}=0.4076.$$

**Fig 1:**
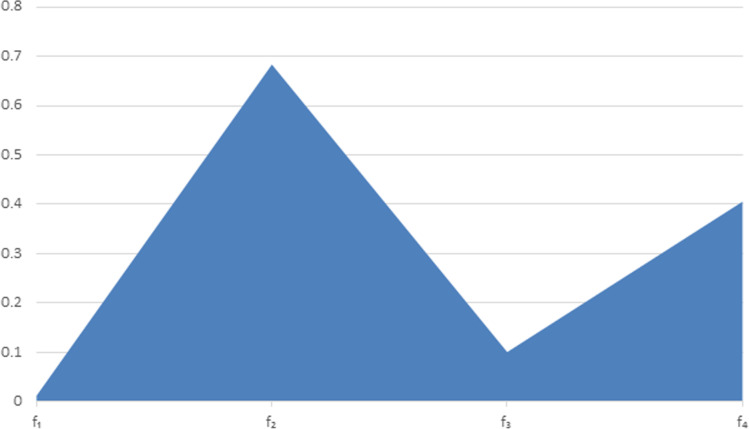
Score function of different ranking.

Step 5: Find the ranking $f_{2}>f_{4}>f_{3}>f_{1}$ and $f_{2}$ is the best ranking.Fig 1 is given as

### 5.2. Comparison analysis

In this design, several sets of criteria weights are characterized by the number of criteria, case we have m criteria, we should define m sets of criteria weights for the affectability examination. In each of the sets, one measure has the most significance (weight), another has the slightest significance, and the other criteria have importance or weights between the foremost and slightest imperative criteria. The weight of each measure in each of the sets is defined in Tables 7.

**Table 7:** comparison method with the existing method.

Operators	$f_{1}(a)$	$f_{2}(a)$	$f_{3}(a)$	$f_{4}(a)$	Ranking
GBNAAWA	0.0126	0.6541	0.1236	0.4456	$f_{2}>f_{4}>f_{3}>f_{1}$
GBNAAOWA	0.6004	0.9097	0.6897	0.5021	$f_{2}>f_{3}>f_{1}>f_{4}$
GBNAAHWA	0.6344	0.5097	0.0007	0.0002	$f_{1}>f_{2}>f_{3}>f_{4}$
GBNAAWG	0.3004	0.4907	0.4567	0.0345	$f_{2}>f_{3}>f_{1}>f_{4}$
NFWG operator [19]	0.4504	0.4758	0.6007	0.4097	$f_{3}>f_{2}>f_{1}>f_{4}$
IFWG operator [14]	−0.0703	−0.0401	−0.0051	0.0987	$f_{4}>f_{3}>f_{2}>f_{1}$

**Fig 2:**
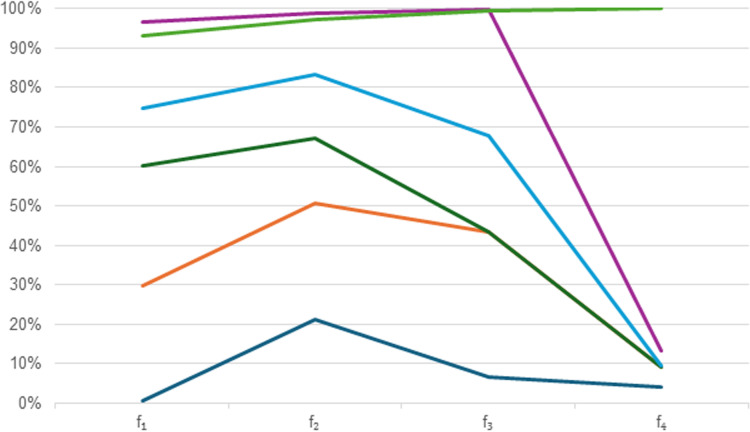
Different rankings of comparison analysis.

Fig 2 is comparison methods as below

NFWG Operator [19]: A thorough description of the NFWG operator’s design, use, and practical implementation. We will also highlight the methodological framework, underlying assumptions, and particular calculations in the original paper that established the NFWG operator.

IFWG Operator [14]: In a similar vein, we will reference the original article for the IFWG operator, outlining its aggregation strategy and any theoretical differences from our approach.

We will also highlight the significant distinctions and parallels between these operators’ techniques, including:

- The function of fuzzy sets in these approaches.
- The computing complexity of the approaches; and
- Any domain-specific presumptions or limitations.

Methodology-Driven Comparison: More than simply numerical results will be included in the updated comparison. The following topics will be covered in detail: • How the expert methodologies (such those employed in [19] and [14]) determine their rankings.

Why The reason why the operators employed in those experiments produce particular ranking results.

- Important distinctions between the approaches, such as how various aggregation techniques (weighted average, harmonic means, etc.) affect the ultimate ranks of the options.

By doing this, we will give the reader a theoretically supported understanding of how our methodology varies from or enhances the expert data.

### 5.3. Sensitivity analysis

In this subsection, we define the sensitivity study in Table 8.

**Table 8:** Sensitivity study.

Operators	Score function	Ranking	Final Ranking
IFS [31]	$\left\{\begin{array}{c} f_{1}=0.0012,\\ f_{2}=0.1204,\\ f_{3}=0.0059,\\ f_{4}=0.1014 \end{array}\right\}$	$\left\{\begin{array}{c} f_{2}>\\ f_{4}>\\ f_{3}>\\ f_{1} \end{array}\right\}$	$\left\{\begin{array}{c} f_{2}>\\ f_{4}>\\ f_{3}>\\ f_{1} \end{array}\right\}$
AAO [32]	$\left\{\begin{array}{c} f_{1}=0.0021,\\ f_{2}=0.1698,\\ f_{3}=0.0139,\\ f_{4}=0.1463 \end{array}\right\}$	$\left\{\begin{array}{c} f_{2}>\\ f_{4}>\\ f_{3}>\\ f_{1} \end{array}\right\}$	$\left\{\begin{array}{c} f_{2}>\\ f_{4}>\\ f_{3}>\\ f_{1} \end{array}\right\}$
NAAO [33]	$\left\{\begin{array}{c} f_{1}=0.0102,\\ f_{2}=0.1409,\\ f_{3}=0.0134,\\ f_{4}=0.0987 \end{array}\right\}$	$\left\{\begin{array}{c} f_{2}>\\ f_{4}>\\ f_{3}>\\ f_{1} \end{array}\right\}$	$\left\{\begin{array}{c} f_{2}>\\ f_{4}>\\ f_{3}>\\ f_{1} \end{array}\right\}$
AAO [34]	$\left\{\begin{array}{c} f_{1}=0.0111,\\ f_{2}=0.3091,\\ f_{3}=0.1202,\\ f_{4}=0.1908 \end{array}\right\}$	$\left\{\begin{array}{c} f_{2}>\\ f_{4}>\\ f_{3}>\\ f_{1} \end{array}\right\}$	$\left\{\begin{array}{c} f_{2}>\\ f_{4}>\\ f_{3}>\\ f_{1} \end{array}\right\}$

### 5.4. Results and discussion

The proposed method starts with major the situation and choosing the fitting specialists, a concern consigned to the executives. This stage is serious, as decision-makers can have variable viewpoints and skills on the theme. The procedure executive allows weights to the specialists’ assessments founded on their contribution or might opt to extravagance all specialists, similarly, supporting with the expectations of the proposed model. The results of this study prove that the proposed decision-making outline, which mixes BNS with aggregations operators and neural schemes, proposes an important progression over existing approaches in the works. The improved aggregation operators GBNAAWA, GBNAAOWA, GBNAAHWA GBNAAWG, GBNAAOWG, and GBNAAHWG demonstrate greater presentation in taking the shades of indecision and fuzziness, which are often incompetently addressed by outmoded fuzzy aggregation approaches. When compared with preceding educations, our results indicate that the proposed outline meaningfully recovers the suppleness and correctness of decision-making in multi-criteria decision-making difficulties.

In this subsection, we introduce the results and discussion in Table 9.

**Table 9:** The results and discussion.

Author	Score function	Ranking	Ranking
RFS [6]	$\left\{\begin{array}{c} f_{1}=0.0006,\\ f_{2}=0.5643,\\ f_{3}=0.1011,\\ f_{4}=0.4098 \end{array}\right\}$	$\left\{\begin{array}{c} f_{2}>\\ f_{4}>\\ f_{3}>\\ f_{1} \end{array}\right\}$	$\left\{\begin{array}{c} f_{2}>\\ f_{4}>\\ f_{3}>\\ f_{1} \end{array}\right\}$
PFS [8]	$\left\{\begin{array}{c} f_{1}=0.0654,\\ f_{2}=0.2278,\\ f_{3}=0.1908,\\ f_{4}=0.2045 \end{array}\right\}$	$\left\{\begin{array}{c} f_{2}>\\ f_{4}>\\ f_{3}>\\ f_{1} \end{array}\right\}$	$\left\{\begin{array}{c} f_{2}>\\ f_{4}>\\ f_{3}>\\ f_{1} \end{array}\right\}$
IVIFS [11]	$\left\{\begin{array}{c} f_{1}=0.0364,\\ f_{2}=0.4636,\\ f_{3}=0.1209,\\ f_{4}=0.4074 \end{array}\right\}$	$\left\{\begin{array}{c} f_{2}>\\ f_{4}>\\ f_{3}>\\ f_{1} \end{array}\right\}$	$\left\{\begin{array}{c} f_{2}>\\ f_{4}>\\ f_{3}>\\ f_{1} \end{array}\right\}$
SM [15]	$\left\{\begin{array}{c} f_{1}=0.0023,\\ f_{2}=0.3274,\\ f_{3}=0.3187,\\ f_{4}=0.3204 \end{array}\right\}$	$\left\{\begin{array}{c} f_{2}>\\ f_{4}>\\ f_{3}>\\ f_{1} \end{array}\right\}$	$\left\{\begin{array}{c} f_{2}>\\ f_{4}>\\ f_{3}>\\ f_{1} \end{array}\right\}$
NS [18]	$\left\{\begin{array}{c} f_{1}=0.1478,\\ f_{2}=0.3654,\\ f_{3}=0.2984,\\ f_{4}=0.3369 \end{array}\right\}$	$\left\{\begin{array}{c} f_{2}>\\ f_{4}>\\ f_{3}>\\ f_{1} \end{array}\right\}$	$\left\{\begin{array}{c} f_{2}>\\ f_{4}>\\ f_{3}>\\ f_{1} \end{array}\right\}$
SVNSs [25]	$\left\{\begin{array}{c} f_{1}=0.0014,\\ f_{2}=0.3201,\\ f_{3}=0.1247,\\ f_{4}=0.1436 \end{array}\right\}$	$\left\{\begin{array}{c} f_{2}>\\ f_{4}>\\ f_{3}>\\ f_{1} \end{array}\right\}$	$\left\{\begin{array}{c} f_{2}>\\ f_{4}>\\ f_{3}>\\ f_{1} \end{array}\right\}$
TM [28]	$\left\{\begin{array}{c} f_{1}=0.0198,\\ f_{2}=0.4325,\\ f_{3}=0.1117,\\ f_{4}=0.1896 \end{array}\right\}$	$\left\{\begin{array}{c} f_{2}>\\ f_{4}>\\ f_{3}>\\ f_{1} \end{array}\right\}$	$\left\{\begin{array}{c} f_{2}>\\ f_{4}>\\ f_{3}>\\ f_{1} \end{array}\right\}$

In addition, the overview of neural schemes, exactly the use of a BN set, enhances the computational competence of the decision-making process. This speech is one of the major limitations in existing approaches, where computational difficulty can often limit their applicability in real-time decision-making schemes. Our outline offers a talented answer for practical requests by balancing computational viability with the essential for precise decision-making.

In standings of theoretic contributions, this study bonds the gap between customary fuzzy set philosophy and contemporary computational intellect techniques, contributing a novel mixture of aggregation operators and neural network-based optimizations. The aptitude to vigorous substitutes additional precisely and compliantly, while management uncertainty and fuzziness, marks an important contribution to the arena of fuzzy decision-making and multi-criteria examination.

### 5.5. Limitation

In this subsection, we define the limitations of the proposed method in Table 10.

**Table 10:** The comparison method with the existing method.

Methods	Best	Normal	Good
[4]	yes	no	no
[5]	yes	no	yes
[6]	yes	yes	yes
[7]	yes	no	yes
[18]	yes	yes	no
[19]	yes	yes	no
[20]	yes	yes	no

Preceding methods, such as individuals using standard bipolar neutrosophic sets or humble weighted averages, frequently fail to completely imprison the multifaceted relations between criteria or to knob indecision successfully. By incorporating aggregation operators, which deliver a universal method of aggregation, our process agrees for the better showing of interdependencies between decision criteria, attracting the decision-making course.

## 6. Conclusion

This paper presents a novel approach to group decision-making under uncertainty through the integration of Aczél-Alsina aggregation operators with generalized bipolar neutrosophic sets (GBNS). The proposed Generalized Bipolar Neutrosophic Aczél-Alsina Weighted Average (GBNAAWA), Generalized Bipolar Neutrosophic Aczél-Alsina Ordered Weighted Average (GBNAAOWA), Generalized Bipolar Neutrosophic Aczél-Alsina Hybrid Weighted Average (GBNAAHWA), Generalized Bipolar Neutrosophic Aczél-Alsina Weighted Geometric (GBNAAWG), Generalized Bipolar Neutrosophic Aczél-Alsina Ordered Weighted Geometric (GBNAAOWG), and Generalized Bipolar Neutrosophic Aczél-Alsina Hybrid Weighted Geometric (GBNAAHWG) operators effectively address complex decision scenarios by handling ambiguity and conflicting information. Through case studies and illustrative examples, we demonstrated the practical applicability and robustness of the proposed method. Comparative analysis showed that the new operators outperform existing methods in terms of decision accuracy, adaptability, and computational efficiency. Sensitivity analysis further highlighted the stability of the approach across various parameter configurations, confirming its potential for widespread application in real-world decision-making problems. Despite its strengths, the proposed method has some limitations, such as the complexity in choosing appropriate weight parameters in certain decision environments. Future research will focus on addressing these limitations, optimizing the operators for larger datasets, and exploring additional decision-making applications. Overall, this work contributes to the advancement of decision-making methodologies, offering a reliable and flexible tool for handling uncertainty in complex group decision-making processes.

In future work, we plan to apply our proposed approach to real-world decision-making problems. Additionally, exploring the potential of Dombi operators, we aim to extend their application to new fuzzy sets, such as linguistic Pythagorean Dombi fuzzy sets and multiplicative sets in [42–44].
